# Dysregulated cellular metabolism drives atherosclerotic plaque progression: a multi-cellular perspective

**DOI:** 10.3389/fcvm.2025.1562707

**Published:** 2025-12-04

**Authors:** Yun-hao Wang, Yu-shan Chen, Si-yu Wang, Bo-yuan Jin, Xin-yi Han, Cheng-jun Hua

**Affiliations:** 1 Heart Center/National Regional (Traditional Chinese Medicine) Cardiovascular Diagnosis and Treatment Center, The First Affiliated Hospital of Henan University of Traditional Chinese Medicine, Zhengzhou, Henan, China; 2 The First Clinical Medical College, Henan University of Traditional Chinese Medicine, Zhengzhou, Henan, China; 3 Collaborative Innovation Center of Prevention and Treatment of Major Diseases by Chinese and Western Medicine, Zhengzhou, Henan, China

**Keywords:** atherosclerosis, macrophages, endothelial cells, vascular smooth muscle cells, metabolic reprogramming

## Abstract

Atherosclerosis (AS) is a chronic inflammatory disease that can lead to severe cardiovascular diseases, primarily characterized by the formation of plaques within arterial walls, resulting in vascular stenosis and hardening. Numerous studies have revealed the complex connection between dysregulated cellular metabolism, specifically metabolic reprogramming, and AS. However, a comprehensive understanding of metabolic reprogramming in AS and its potential as a therapeutic target still requires further exploration. This article provides a comprehensive review of the role of dysregulated cellular metabolism in AS, with a particular focus on the phenomenon of metabolic reprogramming in diseased cells. It discusses in detail the adjustments in lipid and glucose metabolism of macrophages, the metabolic responses of endothelial cells under blood flow shear stress, oxidative stress, and inflammatory stimulation, as well as the metabolic changes of smooth muscle cells during phenotypic transformation. Furthermore, it analyzes how these dysregulated cellular metabolism affect the development of AS. Additionally, the article outlines the mechanisms by which chemically synthesized drugs and Chinese patent medicines treat AS by regulating metabolic pathways, offering a new perspective for disease research and clinical treatment.

## 1 Introduce

Atherosclerosis (AS) is a chronic inflammatory disease characterized by lipid deposition, fibrosis, and calcification in the arterial wall, which leads to arterial lumen stenosis and thrombus formation (1). Currently, the specific pathogenesis of AS has not been fully clarified. The lipid infiltration theory, the endothelial injury-response theory, the platelet aggregation and thrombosis theory, the smooth muscle clone theory, etc. have elaborated it from different perspectives (2). And recent studies have gained a new understanding of the occurrence and development of AS caused by metabolic changes. Metabolic reprogramming is a general term for changes in cellular metabolism, involving changes in multiple metabolic pathways such as those of glucose, lipids, and amino acids. Research has shown that during the course of AS, there are metabolic changes in vascular cells. Therefore, conducting research from the perspective of dysregulated cellular metabolism in vascular cells can lead to a new understanding of the pathogenesis of AS. This article will elaborate on the roles of macrophages, endothelial cells (ECs), and vascular smooth muscle cells (VSMCs) in AS as well as the phenomenon of metabolic reprogramming by respectively analyzing the changes in their metabolism, and explore the mechanism of cellular dysregulated cellular metabolism in AS and their impact on the occurrence and development of AS.

## 2 Metabolic reprogramming

Metabolic reprogramming refers to the process in which cells actively adjust the flow of various metabolites and metabolic patterns in their own metabolic pathways to adapt to changes in the internal and external environment. Under normal circumstances, cells have relatively stable metabolic patterns to maintain functions such as survival, growth, and proliferation. However, when affected by factors such as nutrient deficiency, hypoxia, growth factor stimulation, and disease states, cells will reshape their metabolic pathways and change the production, consumption, and mutual conversion of metabolites.

Metabolic reprogramming was first discovered in the research on tumor cells. The metabolism of tumor cells is significantly different from that of normal cells. For example, in the famous Warburg effect, tumor cells preferentially carry out glycolysis to produce lactic acid even under aerobic conditions, instead of using glucose through the more efficient oxidative phosphorylation, which indicates the reprogramming of the metabolic mode of cells in the diseased state. Subsequently, with the continuous development of research techniques, the phenomenon of metabolic reprogramming in various types of cells has been observed in more physiological and pathological scenarios (3).

## 3 Dysregulated metabolism in macrophages

### 3.1 Changes in lipid metabolism in macrophages

The lipid metabolism of macrophages can be divided into three processes. Firstly, it is lipid uptake. Macrophages take up oxidized low-density lipoprotein (Ox-LDL) in the blood through scavenger receptors such as Cluster of Differentiation 36 (CD36), Scavenger Receptor Class A Member 1 (SR-A1), and Lectin-like Oxidized Low-Density Lipoprotein Receptor-1 (LOX-1).

Secondly, it is lipid metabolism. The ingested lipids are broken down in the lysosomes inside macrophages, releasing free cholesterol and free fatty acids. Free cholesterol can be re-esterified to form cholesterol esters and stored in lipid droplets in the cytoplasm, which is a key step in the formation of foam cells.

Thirdly, it is cholesterol efflux. Macrophages regulate the expression of proteins related to cholesterol efflux by activating specific transcription factors, such as Liver X Receptor (LXR) and Peroxisome Proliferator-Activated Receptors (PPARs). Examples of these proteins include ATP-binding cassette transporter A1 (ABCA1) and ATP binding cassette subfamily G member 1 (ABCG1). These transporter proteins facilitate the transfer of free cholesterol to lipoproteins [especially High-Density Lipoprotein (HDL)], and then form mature HDL particles. This process is a crucial part of the reverse cholesterol transport mechanism (4).

#### 3.1.1 The impact of CD36 on macrophages during lipid uptake

CD36 is a scavenger receptor, mainly expressed on immune cells such as macrophages. It plays a crucial role in the development of AS by mediating the uptake of Ox-LDL, promoting the formation of foam cells and inflammatory responses. After Ox-LDL is taken up by macrophages through the CD36 receptor, specific oxidized lipid components in Ox-LDL (such as 9-HODE and 13-HODE) activate PPARγ, which positively feeds back to upregulate the expression of CD36, increases lipid accumulation in macrophages, and promotes the formation of foam cells (5). Regulatory Factor X1 (RFX1) is a transcription factor. *in vitro*, Yang et al. demonstrated that overexpression or knockdown of RFX1 in mouse peritoneal macrophages (PMAs) and THP-1 cells directly suppresses CD36 transcription, reduces CD36 protein levels, and consequently inhibits oxLDL uptake and lipid accumulation. Extending to an *in vivo* setting, the same team generated myeloid-specific RFX1-knockout *ApoE^−/−^* mice and performed bone-marrow transplantation experiments; loss of RFX1 significantly elevated CD36 expression, increased foam-cell formation, and exacerbated atherosclerotic lesion area, confirming that the RFX1-CD36 axis operates robustly both *in vitro* and *in vivo* (6). In addition, it was found through experiments that Triggering Receptor Expressed on Myeloid Cells 2 (TREM2) also has a regulatory effect on CD36. TREM2 can activate CD36 by regulating Mitogen-Activated Protein Kinase p38 (MAPK-p38) and PPARγ, promoting the lipid accumulation process in macrophages (7). Bao and colleagues constructed bone marrow-specific TREM2 knockout mice and found that after 12 weeks of Western diet feeding, these mice developed smaller aortic root plaques with reduced CD36 expression and fewer Oil Red O-positive foam cells. Administration of pioglitazone, a PPARγ agonist, reversed this protective phenotype, confirming that the TREM2–PPARγ–CD36 signaling axis plays a key role in promoting plaque progression *in vivo* (8).

#### 3.1.2 The role of cholesterol metabolism in macrophages

Cholesterol efflux primarily occurs through two main pathways. The first is the ABCA1 pathway, which is predominantly mediated by Apolipoprotein A-I (apoA1). The second pathway is the ABCG1 pathway, which operates independently of apoA1 and directly transfers cholesterol onto lipoproteins, particularly HDL (9). Research has shown that one of the key factors influencing these pathways is the Liver X Receptor (LXR), a nuclear hormone receptor that senses metabolites. LXR can bind to the promoter elements of the ABCA1 gene and upregulate the expression of the ABCA1 protein, thereby promoting the efflux of cellular cholesterol (10). In cell experiments, homocysteine (Hcy), an independent risk factor for atherosclerosis, was shown to inhibit the LXRα–ABCA1 lipid metabolism pathway in THP-1-derived foam cells by downregulating LXRα, ABCA1, and ABCG1 expression, thereby promoting foam cell formation. This mechanism was further confirmed *in vivo*: *ApoE^−/−^*mice fed a high-methionine diet developed larger plaques and lipid cores with reduced ABCA1 expression in macrophages, effects that were reversed by the LXRα agonist GW3965, demonstrating that Hcy impairs cholesterol efflux through suppression of the LXRα–ABCA1 axis (11).

**Figure 1:**
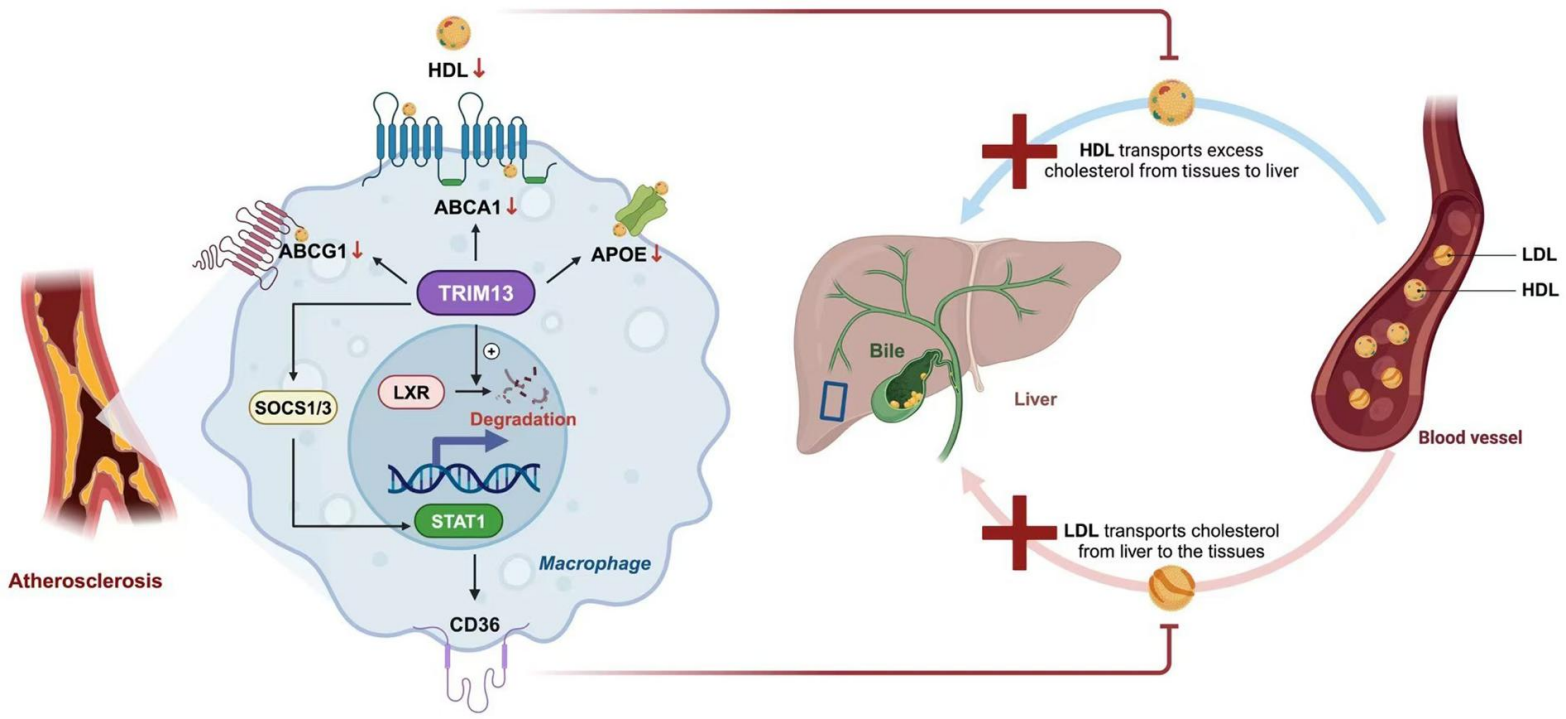
Cholesterol efflux in AS. In macrophages, cholesterol metabolism is primarily mediated by transporters such as ATP-binding cassette transporter A1 (ABCA1), ATP-binding cassette subfamily G member 1 (ABCG1), and apolipoprotein E (ApoE), which facilitate the efflux of cholesterol to high-density lipoprotein (HDL) and contribute to reverse cholesterol transport (RCT) toward the liver. Liver X receptor (LXR) enhances this process by upregulating the expression of ABCA1 and ABCG1. During atherosclerosis, tripartite motif-containing protein 13 (TRIM13) disrupts lipid homeostasis through two major pathways. First, TRIM13 promotes LXR degradation, thereby suppressing the LXR–ABCA1 axis and impairing cholesterol efflux. Second, TRIM13 activates signal transducer and activator of transcription 1 (STAT1) by ubiquitinating and degrading suppressors of cytokine signaling 1 and 3 (SOCS1/3), which increases oxidized low-density lipoprotein (Ox-LDL) uptake via cluster of differentiation 36 (CD36). Together, these effects accelerate foam cell formation and promote atherogenesis.

Recent studies have discovered that during atherosclerosis, the expression of Tripartite Motif Containing 13 (TRIM13) in macrophages increases. TRIM13 is a ring-type E3 ubiquitin ligase that can reduce the expression of ABCA1, ABCG1, and Apolipoprotein E (ApoE) by degrading LXR, thus blocking cholesterol efflux. Additionally, it can regulate CD36. TRIM13 activates Signal Transducer and Activator of Transcription 1 (STAT1) by ubiquitinating and degrading Suppressor of Cytokine Signaling 1/3 (SOCS1/3). The activation of STAT1 enhances the expression of CD36, increases the uptake of Ox-LDL, and promotes the transformation of macrophages into foam cells. Therefore, TRIM13 both inhibits cholesterol efflux and promotes lipid uptake, facilitating the transformation of macrophages into foam cells (12). Importantly, recent *in vivo* studies have confirmed these findings. In *ApoE^−/−^* mice, genetic deletion of Trim13 significantly reduced lipid accumulation, increased ABCA1/ABCG1 expression, and attenuated atherosclerotic plaque development, providing direct evidence that TRIM13 is a critical regulator of cholesterol homeostasis and foam cell formation *in vivo* (12) (Figure 1).

In addition to the above-mentioned processes, intermediates in cholesterol synthesis also exert regulatory effects on macrophages,such as desmosterol, 25-Hydroxycholesterol and squalene.

Desmosterol regulates macrophage function in atherosclerosis by activating LXR signaling to suppress inflammation and enhance cholesterol efflux. Its depletion exacerbates vascular inflammation and disease progression in mouse models, establishing its essential role in metabolic-inflammation balance (13). As the primary endogenous LXR ligand in macrophage foam cells, desmosterol coordinates cholesterol and fatty acid metabolism while suppressing inflammatory gene expression (14). Notably, it selectively activates LXR target genes without triggering SREBP-mediated lipogenic pathways, thereby avoiding adverse effects like hypertriglyceridemia associated with synthetic LXR agonists. These properties position desmosterol as a promising therapeutic target for atherosclerosis treatment (15).

25-Hydroxycholesterol (25-HC), an oxysterol generated by cholesterol 25-hydroxylase (CH25H), exerts pleiotropic effects on macrophage pathophysiology. In animal experiments, studies using myeloid-specific CH25H knockout *ApoE^−/−^* mice showed that loss of CH25H markedly reduced 25-hydroxycholesterol (25-HC) levels and was associated with thicker fibrous caps and greater smooth muscle cell preservation within atherosclerotic plaques. In contrast, exogenous administration of 25-HC (5 mg/kg every other day for 8 weeks) led to fibrous cap thinning and reduced α-smooth muscle actin (α-SMA^+^) cell content, indicating that 25-HC directly compromises plaque stability *in vivo* (16). Mechanistically, 25-HC engages nuclear receptor RORα to reprogram lipid droplet dynamics, balancing macrophage lipid uptake and storage (17). These multifunctional properties establish 25-HC as a critical regulator of inflammatory signaling, lipid homeostasis, and atherosclerotic progression in macrophages.

Squalene exerts dual regulatory functions in macrophage physiology. As an anti-inflammatory agent, it suppresses intracellular reactive oxygen species (ROS), nitrite production, and pro-inflammatory mediators (TNF-α, IL-1β, IL-6, IFN-γ) while downregulating inflammatory enzymes (iNOS, COX-2, MPO). This is achieved through coordinated modulation of TLR4/NF-κB signaling and upregulation of cytoprotective systems including HO-1, Nrf2, and PPARγ. Concurrently, squalene enhances cholesterol homeostasis by activating LXRα/β nuclear receptors, stimulating transcriptional upregulation of ABCA1, ABCG1, and *ApoE^−/−^*critical effectors of reverse cholesterol transport (18). These coordinated actions attenuate macrophage hyperactivation and promote lipid clearance.

#### 3.1.3 The role of fatty acid metabolism in macrophages

The study reveals that the balance between *de novo* fatty acid synthesis (FASN-driven) and β-oxidation (CPT1a-mediated) determines macrophage polarization direction, which may offer novel therapeutic directions and targets for AS.

FASN, a fatty acid synthesis enzyme, also plays a key role in macrophage metabolic regulation and polarization. Cell-based assays demonstrate that FASN is upregulated under specific stimulation, which promotes cholesterol synthesis, activates the Akt-MAPK signaling axis, and thereby increases the expression of M1 macrophage markers; its metabolic product palmitate also provides lipid platforms for NLRP3 inflammasome assembly, leading to excessive reactive oxygen species (ROS) production and enhanced inflammation. In animal experiments, myeloid-specific FASN knockout mice exhibit significantly smaller aortic atherosclerotic plaques, reduced numbers of M1 macrophages, restored mitochondrial oxidative phosphorylation function, and inhibited NLRP3 activation when exposed to a pro-atherosclerotic environment. These findings confirm that FASN-driven lipogenesis serves as a critical metabolic checkpoint in the progression of atherosclerosis (19).

β-oxidation (FAO) significantly affects macrophage metabolism and function through the CPT1A-IL-10 axis. In LPS-induced mouse models, CPT1A, a key FAO enzyme, is downregulated, driving macrophages from an anti-inflammatory M2 to a pro-inflammatory M1 phenotype. This downregulation exacerbates LPS-induced mitochondrial dysfunction, marked by increased ROS and imbalanced mitochondrial fission/fusion proteins, and boosts NLRP3 inflammasome activation and pro-inflammatory cytokine secretion. However, exogenous IL-10 can reverse these effects, underscoring its critical anti-inflammatory role in the CPT1A-IL-10 axis (20).

The different subtypes of fatty acids still play an important role in macrophages. Saturated fatty acids (such as palmitic acid) can activate Toll-like receptor (TLR) 2 and TLR4, generating metabolic products like phosphatidylcholine, diacylglycerol and ceramide. These products then activate protein kinase C (PKC), endoplasmic reticulum stress and reactive oxygen species (ROS) generation pathways, thereby intensifying the inflammatory response. Moreover, palmitic acid can enhance the inflammatory response and cause insulin resistance by up—regulating the expression of fatty acid—binding protein 4 (FABP4/aP2) (21).

Monounsaturated fatty acids (such as oleic acid) have both pro- and anti-inflammatory effects on macrophages. In some cases, oleic acid can inhibit inflammation by reducing palmitic-acid-induced secretion of pro-inflammatory cytokines and NLRP3 inflammasome activation. In other cases, it may enhance inflammation by activating specific signaling pathways. Oleic acid can also suppress the expression of pro-inflammatory molecules by activating microRNA let-7b, while promoting the expression of anti-inflammatory molecules such as mannose receptor C-type 1 (Mrc1), interleukin-10 (IL-10) and transforming growth factor β − 1 (TGF-β1). In addition, oleic acid can influence the inflammatory response by increasing cholesterol efflux in macrophages to regulate lipid metabolism (22).

Polyunsaturated fatty acids (such as ω-3 fatty acids DHA and EPA) exert anti-inflammatory effects through the GPR120 receptor. GPR120 is a G protein-coupled receptor mainly expressed in pro-inflammatory macrophages and mature adipocytes. ω-3 fatty acids can inhibit TLR and TNF-α signaling pathways by activating GPR120, blocking the activation of IKKβ/NFκB and JNK/AP1 signaling pathways, thereby reducing the inflammatory response. Moreover, ω-3 fatty acids can reduce the accumulation of pro-inflammatory M1-type macrophages and increase the expression of anti-inflammatory M2-type macrophages, improving insulin sensitivity. Animal experiments have shown that these anti-inflammatory and insulin-sensitizing effects disappear in GPR120-knockout mice, confirming that their effects are GPR120-dependent (23) (Table 1).

**Table 1:** Effects of different subtypes of fatty acids in macrophages.

Fatty Acid subtype	representative substance	Key pathways/molecules involved	Effects on macrophages	Effects on atherosclerosis
Saturated Fatty Acids	Palmitic Acid	TLR2, TLR4, PKC, FABP4/aP2	Promote inflammation	Promote
Monounsaturated Fatty Acids	Oleic Acid	microRNA let-7b, NLRP3 inflammasome	Dual-effect	Dual-effect
Polyunsaturated Fatty Acids	ω-3 Fatty Acids DHA and EPA	GPR120, TLR, TNF-α,IKKβ/NFκB, JNK/AP1	Inhibit inflammation	Inhibit

TLR, toll-like receptor; PKC, protein kinase C; FABP4, fatty acid–binding protein 4; NLRP3, nucleotide-binding oligomerization domain–like receptor family pyrin domain–containing 3; GPR120, G-protein–coupled receptor 120; DHA, docosahexaenoic acid; EPA, eicosapentaenoic acid.

### 3.2 Glucose metabolism in macrophages

Recently, a research team led by Xuan Wang discovered that the HIF-1α/PFKFB3/NLRP3 axis is a key mechanism for the inflammatory activation of macrophages (24). During the lipid metabolism process in macrophages, Lipopolysaccharide (LPS) binds to Toll-like receptors (TLRs) and activates Hypoxia-inducible factor 1-alpha (HIF-1α) through the Mammalian Target of Rapamycin (mTOR) signaling pathway (25). The activation of HIF-1α induces the expression of 6-phosphofructo-2-kinase/fructose-2,6-bisphosphatase 3(PFKFB3), a key enzyme in glucose metabolism. A team led by Kikkie Poels found through animal experiments and studies on advanced coronary artery plaques that PFKFB3 is positively correlated with plaque vulnerability. Specifically, high expression of PFKFB3 promotes the formation of vulnerable plaques (24, 26). The activation of PFKFB3 provides energy for the NOD-like receptor protein 3 inflammasome (NLRP3) inflammasome, thereby enhancing macrophage inflammation. Consistently, Guo et al. provided strong genetic evidence by demonstrating a gene-dosage effect of Pfkfb3 in *ApoE^−/−^* mice. Both heterozygous deficiency (Pfkfb3^+/−^) and myeloid-specific deletion of Pfkfb3 significantly reduced lesion size, enhanced plaque stability, and shifted macrophage metabolism towards a less glycolytic and more oxidative phenotype. These changes were accompanied by reduced NLRP3 inflammasome activation and diminished pro-inflammatory cytokine release, underscoring the *in vivo* importance of PFKFB3-driven glycolysis in macrophage-mediated atherogenesis (27).

Therefore, inhibiting PFKFB3 can block the process of glycolysis and the activation of the NLRP3 inflammasome, which may become a means to delay the progression of atherosclerosis (28).

In addition, changes in the expression of key enzymes in the tricarboxylic acid (TCA) cycle also play a significant role. Under inflammatory stimulation, Lipopolysaccharide (LPS) leads to a decrease in the expression levels of key enzymes in the TCA cycle, including Citrate synthase (CS), alpha-ketoglutarate dehydrogenase (OGDH), Isocitrate dehydrogenase 2 (IDH2), and Malate dehydrogenase 2 (MDH2), resulting in a decline in TCA cycle metabolism (29). Meanwhile, under LPS stimulation, itaconic acid is produced within macrophages. As an intermediate product in the TCA cycle, itaconic acid can affect Succinate dehydrogenase (SDH) through competitive inhibition. This slows down the conversion of succinate to fumarate, reduces the activity of the electron transport chain, and thus inhibits mitochondrial oxidative phosphorylation, shifting the energy metabolism of macrophages more towards glycolysis (30, 31).

At the same time, during AS, a nuclear receptor in macrophages, Nuclear Receptor 4A1 (NUR77), is also activated. NUR77 can bind to the promoter region of the IDH gene to inhibit its expression. Research has shown that, compared with normal mice, in mice with NUR77 knocked out, the expression of IDH in macrophages increases, enhancing the TCA cycle. This leads to the accumulation of intermediate products such as succinate, promoting the increase in SDH activity and the production of reactive oxygen species (ROS), and enhancing the inflammatory activity of macrophages. This indicates that NUR77 helps to delay the development of atherosclerosis (32).

### 3.3 Amino acid metabolism in macrophages

A growing body of evidence places amino-acid metabolism at the center of macrophage immunometabolic reprogramming in atherosclerosis, with direct consequences for plaque inflammation and stability. The tryptophan (Trp)-kynurenine (Kyn) axis is frequently upregulated in inflammatory lesions: in experimental studies, pharmacological inhibition of IDO1 (e.g., with 1-methyl-tryptophan) in *ApoE^−^/^−^* mice fed a high-fat diet leads to decreased Kyn/Trp ratio, reduced plaque area, and lower macrophage infiltration, indicating that IDO1 activity contributes to lesion development *in vivo* (33). Conversely, mice lacking Ido1 show altered calcification and vascular responses, although the effect on atherogenesis is complex and context-dependent (34). More recently, intestinal epithelial cell-specific deletion of Ido1 in *Ldlr^−^/^−^* mice under HFD+HCD resulted in increased systemic inflammation, augmented lesional T-cell accumulation, and larger atherosclerotic plaques, indicating that tissue-specific IDO1 modulation can influence systemic and vascular immune states (35).

Parallel work has highlighted glutamine/glutamate metabolism as a key substrate network in lesion macrophages. In mice, genetic or pharmacologic inhibition of glutaminase (GLS) impairs macrophage glutaminolysis, reduces the supply of α-ketoglutarate to the TCA cycle, and limits ROS and NAD(P)H generation required for inflammatory effector functions and efferocytosis; such interventions attenuate plaque inflammation and shift lesion composition (e.g., lower macrophage necrotic core) in preclinical models (36).

Finally, arginine metabolism remains a pivotal bifurcation *in vivo* that shapes macrophage phenotype during atherogenesis. In several studies, overexpression of inducible nitric oxide synthase (iNOS) in macrophages enhances NO production, supports classical activation and inflammatory responses; in contrast, enhancing arginase-1 (ARG1) activity (which diverts arginine toward ornithine and polyamines) supports reparative macrophage programs and improves efferocytosis. In murine models, pharmacologic arginase inhibition has been shown to exacerbate vascular injury and impair M2 macrophage accumulation, whereas strategies favoring ARG1 over iNOS shift the balance toward more stable plaques (37).

## 4 Endothelial cell metabolism in atherosclerosis

### 4.1 The impact of wall shear stress on glucose metabolism of endothelial cells

Glycolysis is the primary pathway for ECs to obtain energy and maintain the integrity and function of the vascular endothelium (38). Recent studies have found that blood flow shear stress is a key factor affecting the glycolysis process (39). It has been observed that in ECs under laminar flow, the 13C labeling of intermediate products of glucose metabolism decreases, indicating that the glycolytic flux of ECs is reduced under laminar flow (40). Subsequent studies have found that laminar shear stress affects the glycolysis process of vascular ECs by activating signaling molecules such as PI3K/Akt, Endothelial Nitric Oxide Synthase (eNOS), and Kruppel-like Factor 2(KLF2) (41). The main signaling molecule at work here is KLF2. Research shows that when ECs are subjected to laminar shear stress, the transcription factor KLF2 is upregulated. KLF2 can increase the expression of eNOS to promote the production of nitric oxide (NO), thus protecting ECs (42). KLF2 also downregulates the expression of key glycolytic enzymes, such as PFKFB3, Hexokinase 2(HK2), and Phosphofructokinase-1(PFK-1), through specific DNA-binding sequences, thereby inhibiting the metabolic process of ECs. This results in a reduction in glucose uptake and mitochondrial content in ECs, ensuring that cell metabolism remains quiescent and thus playing a protective role against atherosclerosis (43).

**Figure 2:**
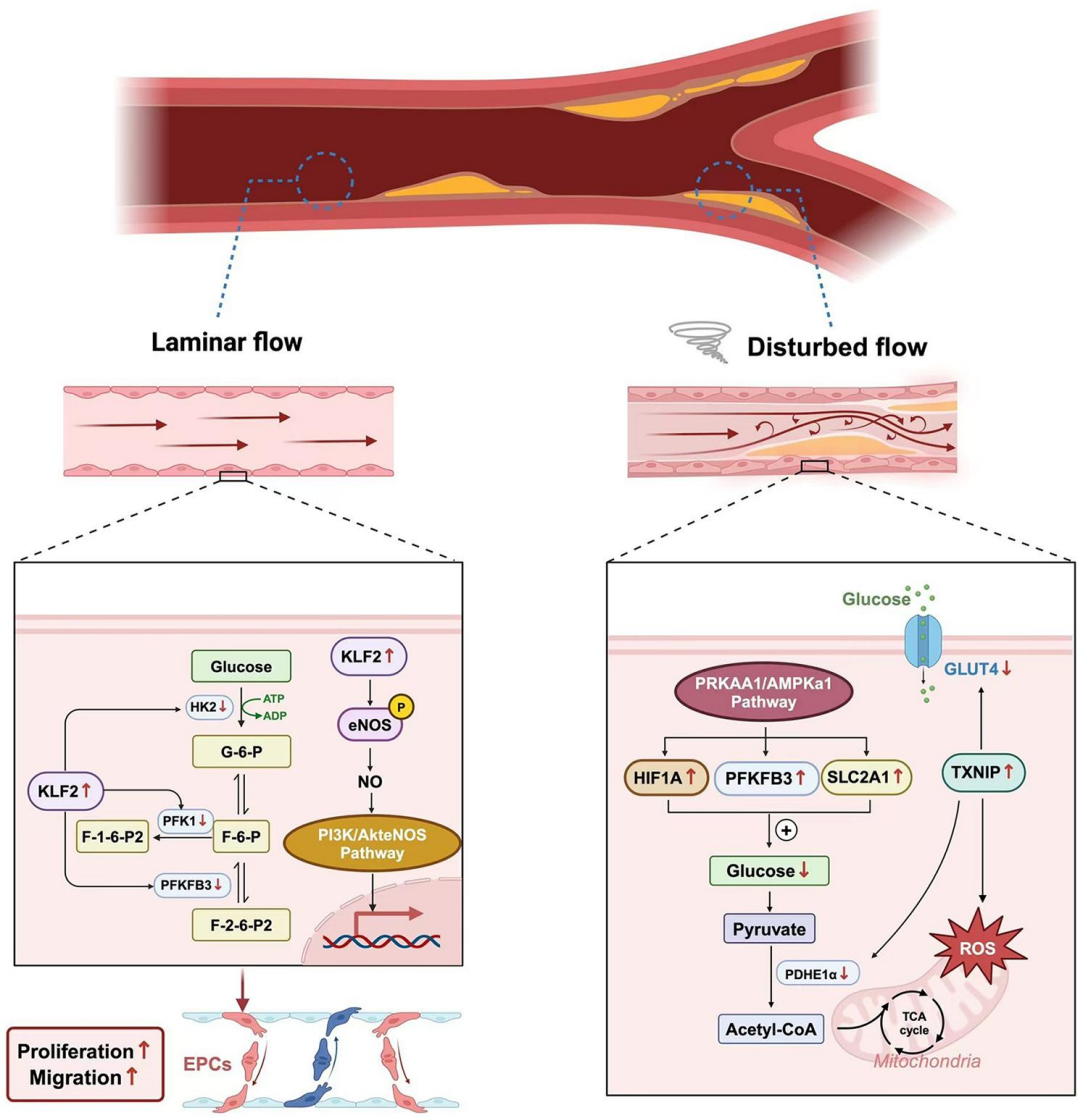
The different effects of wall shear stress.

In regions with disturbed blood flow, turbulence promotes the glycolysis process by activating the PRKAA1/AMPKα1 signaling pathway and upregulating the expression of glycolysis-related genes, such as HIF1α, Solute Carrier Family 2 Member 1(SLC2A1), and PFKFB3 (44). Meanwhile, *in vitro* experiments have shown that turbulence can lead to an increase in the expression of Thioredoxin-interacting Protein (TXNIP) in human ECs (45). The study found that the level of mitochondrial ROS decreased after treatment with a TXNIP inhibitor, indicating that TXNIP causes mitochondrial dysfunction under the induction of turbulence, resulting in damage to endothelial function. Wang et al. used a mouse carotid artery partial ligation model and found that the expression of TXNIP in the low shear stress region was significantly increased, accompanied by excessive mitochondrial ROS, as well as decreased activities of GLUT4 and PDH. Administration of a TXNIP inhibitor could reverse such metabolic disorders and restore endothelial function (46) (Figure 2).

Recent studies have revealed a new mechanism of the effect of turbulence on ECs. A team led by Yifei Lv found that low shear stress can activate Inhibitor of Nuclear Factor Kappa-B Kinase Epsilon (IKKε), which in turn promotes the phosphorylation and nuclear translocation of STAT1. The activated STAT1 further binds to the promoter region of the NLRP3 gene, enhancing the expression of NLRP3. This process leads to pyroptosis of ECs, clarifying for the first time the new mechanism by which low shear stress affects ECs through the IKKε/STAT1/NLRP3 signaling pathway (47).

Shear stress in blood vessels refers to the frictional force exerted by flowing blood on the vascular wall, which profoundly influences ECs metabolism, structure, and function. Two major patterns of shear stress exist: laminar shear stress and disturbed (oscillatory) shear stress. Laminar shear stress, typically found in straight arterial segments, acts parallel to the vessel axis and exerts protective effects on ECs by upregulating Kruppel-like factor 2 (KLF2). KLF2 suppresses glycolysis-related enzymes such as phosphofructokinase-1 (PFK-1), phosphofructokinase-2/fructose-2,6-bisphosphatase 3 (PFKFB3), and hexokinase 2 (HK2), while enhancing endothelial nitric oxide synthase (eNOS) expression to promote nitric oxide (NO) production and vascular homeostasis. In contrast, disturbed shear stress, generated at arterial bifurcations or regions of turbulent flow, activates AMP-activated protein kinase alpha 1 (AMPKα1) and thioredoxin-interacting protein (TXNIP), which disrupt mitochondrial function, increase reactive oxygen species (ROS) generation, and impair glycolytic balance. These changes collectively induce endothelial dysfunction and promote the initiation and progression of AS (Figure 2).

### 4.2 The impact of oxidative stress and inflammatory stimulation on glucose metabolism

Mitochondria are the primary sites for glycolysis and oxidative phosphorylation in ECs. Oxidative stress regulates glycolysis in ECs by influencing mitochondrial function. When Protein kinase C beta II (PKCβII) in ECs is activated, it leads to the phosphorylation of the p66Shc adaptor protein (p66shc) at the Serine36 site. p66shc is a mitochondrial protein, and its phosphorylated form increases the production of mitochondrial ROS. Long-term or excessive increases in ROS can result in mitochondrial dysfunction, affecting the normal functions of mitochondria and thus inhibiting the glycolysis process in ECs (48). Under hypoxic conditions, HIF1α helps ECs absorb glucose as a substrate for glycolysis by increasing the expression of GLUT1. Additionally, HIF1α can upregulate the expression of key glycolytic enzymes, thereby promoting the glycolysis process (49).

The enhancement of inflammatory stimulation disrupts the glycolysis process in ECs. Under inflammatory conditions, the energy production mode of ECs shifts from oxidative phosphorylation to glycolysis to meet the energy demands during the inflammatory process. Johan G. Schnitzler demonstrated through cell and animal experiments that LPS activates ECs through its oxidized phospholipids (OxPLs), causing ECs to express more inflammatory factors. Meanwhile, OxPLs upregulate the glycolysis process in ECs by promoting the expression of PFKFB3, providing additional energy and metabolites to support the inflammatory response and cell proliferation, and thus promoting the progression of AS (50).

### 4.3 Lipid metabolism of endothelial cells

Through enzyme-linked immunosorbent assay (ELISA), researchers found that the content of endothelial lipase (EL) increases in AS (51). EL can affect ECs through two pathways: by hydrolyzing HDL and by influencing the adhesiveness of ECs. By comparing mice with the EL gene knocked out and control group mice, researchers found that the HDL-C content in the EL knockout mice was significantly higher than that in the control group mice. This indicates that EL can regulate the lipid metabolism of ECs by affecting HDL-C (52). Additionally, EL affects ECs by influencing their adhesiveness. EL can promote the synthesis of interleukin-8 (IL-8) in ECs, which in turn upregulates the expression of intercellular adhesion molecule 1 (ICAM-1) (53). This causes inflammatory factors and cells to gather around the lesion and damage ECs, thereby promoting the development of AS (54).

The latest research has focused on sphingolipid metabolism. It has been found that the early remodeling of ECs metabolism caused by changes in hemodynamics is conducive to the activation of the sphingosine-1-phosphate (S1P) signal rather than the signaling pathway of amide substances. This finding overturns the traditional view that the accumulation of amide substances leads to ECs dysfunction. Moreover, the deletion of reticulon 4B (NOGO-B) helps maintain the balance of sphingolipid metabolism, making it tend to generate sphingosine-1-phosphate (S1P) rather than ceramide. This metabolic tendency helps protect ECs from the negative impacts of hemodynamic changes and can inhibit the development of coronary atherosclerosis. These findings provide a new direction for sphingolipid-based therapies and help offer new targets for the treatment of AS (55).

Triglyceride (TG) metabolism is closely related to the occurrence and development of AS. Triglyceride-rich lipoproteins (TRL), composed mainly of chylomicrons (CM) from the intestine and very-low-density lipoproteins (VLDL) from the liver, are a type of lipoprotein rich in TG. Studies show that TRL affects ECs through multiple mechanisms. Specifically, TRL can induce the expression of adhesion molecules such as ICAM-1, VCAM-1, and various selectins on ECs. This increases the adhesion of WBCs to ECs and promotes their migration into the subendothelial space, thereby contributing to atherosclerotic lesions (56). TRL can also lead to endothelial dysfunction (ED), impairing the normal physiological functions of ECs, such as regulating vascular tone, preventing thrombosis, and reducing inflammation. This compromises the vascular endothelial barrier, increases vascular permeability, and facilitates the entry of lipoproteins and inflammatory cells into the subendothelial space. In addition, TRL activates various inflammatory and oxidative stress (OS) signaling pathways, releases oxidized FFAs, and stimulates the expression of inflammatory cytokines by WBCs (such as neutrophils, T cells, and monocytes), triggering inflammatory responses in the arterial wall (57). TRL can induce the production of ROS such as superoxide anions, hydroxyl radicals, and peroxynitrite in ECs, increasing OS, causing endothelial cell damage and death, and further promoting WBC adhesion and inflammatory responses. TRL also induces endothelial cell apoptosis through the secretion of pro-apoptotic cytokines (such as TNF-α and IL-1β), thereby exacerbating vascular damage and AS. Moreover, TRL affects the response of ECs to blood components, promoting platelet aggregation and coagulation by facilitating the assembly of the prothrombinase complex and upregulating the expression of plasminogen activator inhibitor-1 (PAI-1). It also upregulates the expression of tissue factor (TF) on ECs, a key initiator of the coagulation cascade. Finally, TRL reduces the anti-AS and anti-inflammatory effects of HDL in ECs, thereby lowering its protective role on blood vessels (58).

Recent studies have shown that in ECs (59), the absence of adipose triglyceride lipase (ATGL) leads to the accumulation of TG and lipid droplets (LD), triggering endoplasmic reticulum (ER) stress, activating the NF-κB signaling pathway, and upregulating pro-inflammatory factors such as VCAM1. At the same time, it reduces the expression of endothelial nitric oxide synthase (eNOS), decreases the synthesis of NO, and impairs endothelium-dependent vasodilation. Animal experiments have demonstrated that in endothelial-specific ATGL knockout mice (Atgl ECKO), there is significant lipid accumulation in pulmonary and aortic ECs, with increased VCAM1 expression in aortic vulnerable areas. When crossed with *ApoE* knockout mice, these mice exhibit enlarged atherosclerotic plaque areas and increased macrophage infiltration under a high-fat diet. Single-cell sequencing confirms the upregulation of early ER stress and inflammation-related genes in ECs (60, 61).

### 4.4 Amino acid metabolism of endothelial cells

**Figure 3:**
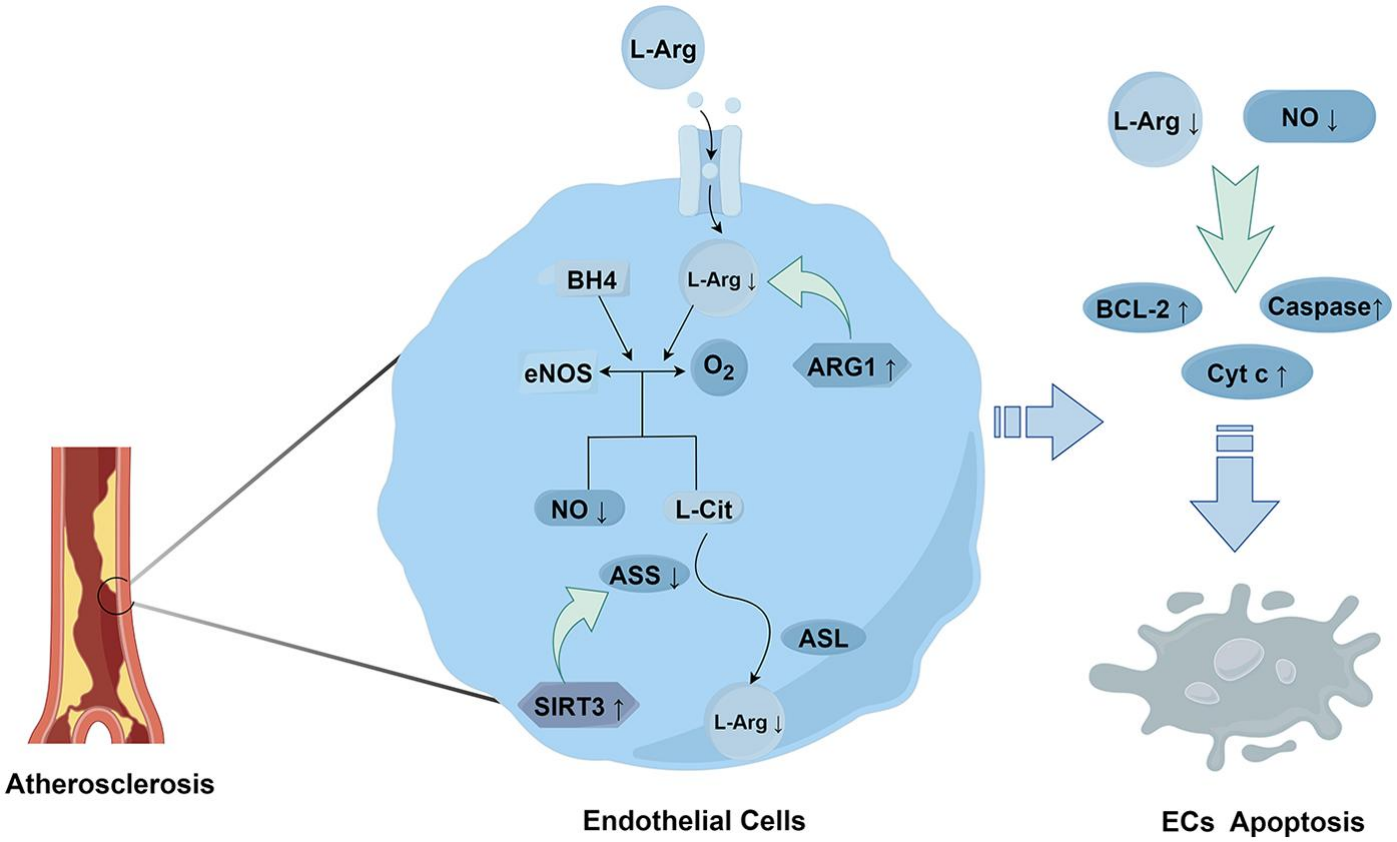
The metabolism and effects of L-arginine.

Amino acid metabolism also plays a role in regulating the functions of ECs. Arginine affects the homeostasis of ECs through the regulation of eNOS. Studies have shown that the activity of eNOS and the production of NO can be influenced by adjusting the concentration of the substrate L-arginine (L-Arg). During the progression of AS, the content of arginase increases (62). Arginase in ECs can break down L-Arg, thereby reducing the amount of L-Arg available for eNOS to synthesize NO. This prevents eNOS from effectively synthesizing NO and leads to vascular endothelial dysfunction (63). Moreover, it has been found that Sirtuin 3 (SIRT3) can regulate argininosuccinate synthase (ASS) through deacetylation, thus affecting the synthesis of arginine. In mice with SIRT3 gene knockout or inhibition, ASS becomes over-acetylated and its activity decreases, resulting in a decline in the level of L-Arg. This further leads to a reduction in NO production, an increase in vascular inflammation, and ultimately promotes the development of AS (64) (Figure 3).

ECs can express phosphoglycerate dehydrogenase (PHGDH). PHGDH is a key enzyme for serine synthesis and affects the serine synthesis pathway (SSP). It was found through silencing PHGDH that a decrease in serine level leads to a reduction in heme biosynthesis. Heme is an important prosthetic group in the cytochromes of the respiratory chain. The lack of heme causes dysfunction of the electron transport chain (ETC) complexes III/IV, resulting in impaired mitochondrial respiration, an increase in reactive ROS production, and enhanced oxidative stress (65). Meanwhile, PHGDH can also delay the aging of ECs through two pathways. One is by promoting serine biosynthesis. Serine, as a co-activator, stimulates the activity of pyruvate kinase M2 (PKM2), which is a key enzyme that can maintain ECs proliferation and vascular integrity. The other is by promoting the stability and nuclear localization of PKM2. PKM2 phosphorylates H3T11 and regulates the expression of aging-related genes (66). Through cell experiments, it was found that serine can also have antioxidant and cytoprotective effects on ECs by inducing other antioxidant factors such as nuclear factor E2-related factor 2 (Nrf2), heme oxygenase 1 (HO-1), and NO (67). In animal experiments, Vandekeere et al. used endothelial-specific PHGDH knockout mice and found that impaired serine synthesis led to a significant decrease in heme levels and the activities of respiratory chain complexes III/IV, along with excessive ROS production and induced endothelial senescence. Supplementation of serine could reverse the aforementioned defects, confirming *in vivo* that serine is capable of maintaining the metabolic homeostasis and functional integrity of vascular endothelium (65).

L-Arginine enters ECs through specific transporters, such as the cationic amino acid transporter 1 (CAT-1). Within ECs, eNOS uses L-arginine and the essential cofactor tetrahydrobiopterin (BH4) as substrates to react with oxygen, generating nitric oxide (NO) and L-citrulline (68). Subsequently, through the actions of argininosuccinate synthase (ASS) and argininosuccinate lyase (ASL), ECs convert L-citrulline (L-Cit), a by-product of NO generation, back to L-arginine. This maintains the levels of L-arginine and ensures the continuous production of NO (69). However, Arginase 1 (ARG1) directly degrades L-arginine, and SIRT3 acts on ASS, preventing L-Cit from being converted back to L-arginine. This leads to a decrease in the levels of NO and L-arginine, resulting in a reduction in the expression of anti-apoptotic proteins such as Bcl-2 and an increase in the activities of apoptotic factors such as cytochrome C and caspases, causing apoptosis in endothelial cells (70).

## 5 Smooth muscle cell metabolism in atherosclerosis

### 5.1 Phenotypic switch of smooth muscle cells

The phenotypic switch of smooth muscle cells refers to the process in which VSMCs change from one phenotype to another under physiological or pathological conditions (71). Under physiological conditions, VSMCs typically exhibit a contractile phenotype, expressing abundant contractile proteins to maintain the normal structure and function of blood vessels, while their proliferation and migration abilities are relatively weak. Under pathological conditions, such as vascular wall injury, growth factor stimulation, or damage from inflammatory factors and ROS, VSMCs respond to stimuli from the surrounding environment and transform from the contractile phenotype to the synthetic phenotype. Synthetic VSMCs adopt an epithelial-like morphology, with decreased expression of contractile proteins and increased expression of synthetic proteins. They possess higher proliferation and migration abilities and can synthesize a large amount of extracellular matrix (72). Additionally, VSMCs can acquire characteristics of other cell types, such as transforming into osteoblast-like, fibroblast-like, or macrophage-like foam cells. For example, inflammatory stimulation or lipid accumulation may cause VSMCs to transform into macrophage-like cells. These cells begin to express macrophage and mesenchymal stem cell markers, exhibit phagocytic activity, and ultimately engulf lipids to become foam cells (73).

### 5.2 The influence of metabolism on the phenotypic switch of smooth muscle

#### 5.2.1 Glucose metabolism

GLUT1, the main glucose transporter in VSMCs, is vital for high glucose utilization and lactate production under normal conditions (74). After vascular injury, VSMCs accumulate in the neointima with increased GLUT1 expression, allowing more glucose to enter cells and enhance lactate and acetyl-CoA formation (56, 75). Increased GLUT1 expression also induces VSMCs proliferation and reduces apoptosis by altering inhibitory protein expression like c-FLICE and c-FLIP (57, 76). In the pentose phosphate pathway, 6PGD impacts VSMCs phenotypic switch. *in vitro*, activating 6PGD via dCas9- exacerbates VSMCs phenotypic switch, suggesting 6PGD can influence VSMCs by activating the downstream ERK/MEK signaling pathway (77, 78), promoting AS progression. Lu further confirmed through animal experiments that 6PGD is a key metabolic switch for VSMCs phenotypic switching: specifically, in mice with smooth muscle-specific 6PGD overexpression or knockout, 14 days after surgery, the overexpression group showed increased neointimal area, decreased expression of the contractile marker α-SMA, and elevated expression of the synthetic marker OPN, while 6PGD^SMKO mice exhibited reduced neointimal area and preserved contractile phenotype. Mechanistically, elevated 6PGD promotes the synthesis of NADPH and nucleotides, maintains a high cellular redox potential, thereby inducing the phosphorylation and activation of ERK1/2 and MEK1, and the MEK inhibitor PD0325901(a selective MEK1/2 inhibitor) can reverse 6PGD-mediated phenotypic switching, confirming that the 6PGD–ERK/MEK axis is the functional core of this regulatory process (77).

The KLF4-PFKFB3 axis is crucial for smooth muscle phenotypic switch. KLF4, key in maintaining embryonic stem cell pluripotency and somatic cell reprogramming (79), enhances glycolytic conversion by upregulating PFKFB3 expression. In experiments, inhibiting glycolysis suppresses smooth muscle phenotypic switch, showing enhanced glycolysis is essential for this switch (80). Cao's team found HIF-1α-PFKFB3 also affects the switch. Mouse experiments show HIF-1α and PFKFB3 induction in neointimal VSMCs. HIF-1α overexpression increases glycolysis, reduces the contractile phenotype, and promotes VSMCs proliferation (81). Thus, PFKFB3 is important in smooth muscle phenotypic switch, and regulating PFKFB3 to inhibit VSMCs phenotypic switch may be a potential therapeutic target for AS.

#### 5.2.2 Lipid metabolism

Excessive intracellular cholesterol and triglycerides can induce smooth muscle cells to transform into the synthetic phenotype due to lipid accumulation altering the intracellular metabolic environment and affecting cell signaling pathways (82). The impact of cholesterol on this phenotypic switch mainly occurs in three aspects.

Firstly, in lipid uptake and receptor regulation, cholesterol-related lipoproteins (such as LDL) are taken up by smooth muscle cells via multiple receptors. Low-Density Lipoprotein Receptor-Related Protein 1(LRP1) can mediate the uptake of aggregated low-density lipoprotein (agLDL), and SR family receptors (such as SR-AI/II, CD36, LOX-1) have a high affinity for modified cholesterol lipoproteins, prompting smooth muscle cells to transform into foam cells after uptake (83, 84).

Secondly, alterations in the intracellular cholesterol metabolic balance involve cholesterol esterification and hydrolysis, as well as the regulation of related enzymes and transporters. Acyl coenzyme A-cholesterol acyltransferase (ACAT) esterifies excess intracellular Free cholesterol (FC) into Cholesterol esters (CE), promoting CE lipid droplet accumulation within the cell and smooth muscle cell transformation into foam cells (85). ACAT-1 is the ACAT isoenzyme in human VSMCs. Animal experiments have demonstrated that inflammatory factors, by activating the TLR4/MyD88/NF-κB pro-inflammatory pathway, can increase ACAT −1 expression in cultured mouse VSMCs and in the atherosclerotic lesions of *ApoE^−/−^* mice, thereby promoting foam cell formation (86). Cholesterol hydrolysis mainly occurs through the interaction between Lysosomal acid lipase (LAL) and Neutral cholesterol ester hydrolase (NCEH). LAL prevents excessive lipid accumulation, and NCEH hydrolyzes excess CE into FC for efflux (87, 88). Maria Franca Mulas and colleagues found that PBMCs from patients with atherosclerosis or familial hypercholesterolemia showed enhanced cholesterol esterification, increased lipid accumulation, and reduced Cav-1, n-CEH, and ABCA1 expression compared with controls. In cultured PBMCs and VSMCs, growth stimulation promoted cholesterol esterification, while RAD treatment reversed these effects and inhibited proliferation. These results suggest that n-CEH/Cav-1–regulated cholesterol esterification serves as a key metabolic checkpoint controlling vascular cell proliferation and phenotype switching in atherosclerosis (89). The balance between esterification and hydrolysis determines the relative content of intracellular FC and CE. When esterification predominates, CE accumulation increases, promoting the foam cell phenotype. When hydrolysis is stronger, FC content increases, which may be more conducive to cholesterol efflux and maintaining the normal cell phenotype. Altering this balance is crucial for the phenotypic switch and functional regulation of vascular smooth muscle cells during pathological processes like AS (90). Joshua A. Dubland's team found that in human and mouse VSMCs, LAL expression and activity are low. This key hydrolytic enzyme defect causes insufficient FC production. It can neither effectively suppress cholesterol synthesis nor activate efflux proteins like ABCA1. Under lipid-accumulation conditions, the balance between hydrolysis and esterification is disrupted, making VSMCs more prone to transforming into foam cells (91).

Thirdly, impaired cholesterol efflux impacts the cell phenotype. Normally, ABCA1, ABCG1, and SR-BI are involved in cholesterol efflux. Cellular experiments have revealed that ABCA1 knockout significantly reduces apoAI-mediated cholesterol efflux, leading to enhanced transdifferentiation of VSMCs into macrophage-like cells (MLCs). In contrast, ABCG1 knockout only partially impairs HDL-mediated cholesterol efflux, as upregulated scavenger receptor BI (SR-BI) compensates for the deficiency, highlighting the critical role of ABCA1/ABCG1 in regulating cholesterol metabolism and phenotypic switching *in vitro* (92). Recent studies have shown that in high-fat diet-induced mouse models, SMC-specific LXR deficiency leads to a significant increase in VSMCs-derived transitional cells (expressing Vcam1 and Ly6a) and a decrease in contractile VSMCs (expressing Acta2 and Myh11). It concurrently downregulates the expression of the cholesterol efflux gene Abca1 and fatty acid synthesis genes Srebf1/Scd1 in VSMCs, disrupting lipid homeostasis. Furthermore, it enhances endoplasmic reticulum (ER) stress and apoptosis in VSMCs, and promotes the high expression of pro-inflammatory and fibrochondrocyte-related genes in transitional cells, ultimately resulting in the instability of atherosclerotic plaques in the aortic root and brachiocephalic artery (93).

However, recent *in vivo* studies using smooth muscle cell-specific ABCA1/ABCG1 double-knockout mice have shown that aortic smooth muscle cells exhibit increased cholesteryl ester (CE) accumulation without triggering endoplasmic reticulum stress or transdifferentiation. Importantly, the size and composition of AS plaques in the aortic root and brachiocephalic artery remain unchanged (94).

The discrepancies between *in vitro* and *in vivo* results likely reflect several factors. First, many *in vitro* studies use immortalized VSMCs lines (such as MOVAS) or isolated primary cells that lack the complex multicellular interactions, extracellular matrix, haemodynamic forces and inflammatory milieu of the arterial intima, which can exaggerate cell-autonomous effects on cholesterol handling and phenotypic switching (92). Second, VSMCs *in vivo* exhibit vessel- and layer-specific lipid handling: aortic/medial SMCs display robust ACAT activity and cholesteryl-ester sequestration that buffer excess free cholesterol, limiting ER stress and transdifferentiation—mechanisms that are not always reproduced in culture. Third, lineage-tracing and single-cell studies show that only subsets of medial VSMCs clonally expand and adopt macrophage-like, fibroblast-like or osteochondrogenic phenotypes within plaques, so perturbing a single pathway across all VSMCs may yield only modest net effects at the lesion level (95). Importantly, recent SMC-specific Abca1/Abcg1 knockout mice accumulated free cholesterol in SMCs but did not develop larger or compositionally different aortic-root or brachiocephalic lesions, a finding attributed to low baseline ABCA1/ABCG1 expression in intimal SMCs and to *in vivo* cholesterol sequestration/compensation (94). Finally, compensatory routes of HDL-mediated efflux (for example, SR-BI upregulation in VSMCs-derived macrophage-like cells) can preserve cholesterol export when ABC transporters are reduced, blunting the impact observed in cell culture. Together, these points explain why robust, cell-autonomous changes in cultured VSMCs (such as altered ABCA1/ABCG1) do not always translate into clear changes in plaque burden or composition in intact animal models.

#### 5.2.3 Amino acid metabolism

VSMCs undergo phenotypic switching from a contractile to a synthetic (or more plastic) state during atherosclerosis, which imposes elevated demands not just for energy but for amino acid–derived substrates to support proliferation, migration, ECM deposition, and survival. Recent *in vivo* and *in vitro* work reveals several amino acid metabolic pathways that underpin these processes.

Firstly, arginine metabolism via Arginase-II (Arg-II) has been demonstrated to promote VSMCs senescence and apoptosis in mouse models. For example, in *ApoE^−^/^−^* mice, genetic ablation of Arg-II significantly reduced VSMCs apoptosis and senescence in the aortic root plaques compared to wild-type controls; this was accompanied by lowered activation of the signaling axes S6K1-JNK-p66Shc, ERK-p66Shc, and p53, suggesting that Arg-II not only modulates arginine catabolism but triggers mitochondrial dysfunction and oxidative stress to drive lesion vulnerability (96).

Secondly, glutamine metabolism appears important for VSMCs survival and phenotypic functions. A study of mouse abdominal aortic aneurysm (AAA) [Angiotensin II+Ca₃(PO_4_)_2_ induced] in *ApoE^−^/^−^* mice showed that glutamine supplementation reduced incidence of aneurysm, preserved elastic fiber architecture, reduced collagen over-deposition, diminished ROS and MMP (matrix metalloproteinase) expression, and decreased VSMCs apoptosis *in vivo*. These findings suggest glutamine protects VSMCs from oxidative stress–, ECM-degradation–, and apoptosis-induced damage in disease models (97).

Thirdly, in cultured VSMCs, glutamine uptake and glutaminase (GLS1) activity support proliferation, migration, and survival. *in vitro* studies (rat or human aortic SMC) show that glutamine increases cell proliferation and migration, that GLS1 inhibitors (e.g., BPTES, CB-839) suppress these responses, and that addition of cell-permeable α-ketoglutarate can partly rescue the effects of glutamine deprivation. While these are *in vitro*, they point to glutamine-dependent supply of TCA cycle intermediates as vital in synthetic VSMCs phenotype (98).

Finally, cross-talk via nitric oxide/arginine pathways also appears in VSMCs. In studies with native LDL stimulation of VSMCs, arginase inhibition (by compounds like limonin) increased intracellular L-arginine levels, suppressed NADPH oxidase–derived ROS, inhibited PKCβII activation, and reduced proliferation. While many of these are cell culture, the arginase expression/activity is increased in lesions, supporting that *in vivo* VSMCs likely experience shifts in arginine handling that impact oxidative stress and growth (99).

## 6 Crosstalk of macrophages, endothelial cells, and vascular smooth muscle cells in metabolic reprogramming

The initiation and progression of atherosclerosis result from the interplay of multicellular metabolic networks, in which ECs, MACs, and VSMCs form a critical “three-cell circuit” Disturbed blood flow (atheroprone flow) and oxidative stress markedly alter EC metabolic states. Recent studies demonstrated that unstable shear stress promotes N6-methyladenosine (m^6^A) modification mediated by METTL3, thereby upregulating glycolytic genes such as PFKFB3 and HK1, which enhances glycolysis, inflammatory signaling, and endothelial permeability (100). Conversely, under laminar flow conditions, KLF2 represses PFKFB3 expression, reduces glycolysis, and maintains vascular homeostasis (101). These metabolic changes impair EC barrier integrity, facilitating LDL penetration into the intima and upregulating adhesion molecules (e.g., VCAM-1, ICAM-1), which promote monocyte adhesion and transmigration (102).

Once infiltrated into the intima, monocytes differentiate into MACs and, under the influence of oxidized LDL, become foam cells. Beyond cholesterol ester accumulation, MACs release inflammatory mediators and metabolites such as 25-hydroxycholesterol, which have been shown in animal models to exacerbate vascular inflammation and promote plaque destabilization (16). Meanwhile, VSMCs undergo phenotypic switching under lipid overload and inflammatory conditions. A recent study of human coronary arteries demonstrated that lipid infiltration drives VSMCs toward a macrophage-like phenotype, characterized by CD68 and FABP4 expression, thereby expanding the foam cell pool (103). Lineage-tracing experiments in *ApoE^−/−^* mice further confirmed that VSMCs can transdifferentiate into foam-like cells, significantly influencing plaque structure and stability (104).

At the molecular level, VSMCs transdifferentiation is accompanied by metabolic reprogramming. For instance, the c-Fos/LOX-1 pathway promotes lipid uptake and foam cell formation via mitochondrial ROS production in response to oxidized LDL; inhibition of c-Fos or ROS scavenging attenuates this process (105). Moreover, Dickkopf-1 (DKK1) has been shown to downregulate ABCA1 through the C/EBPδ–CYP4A11–SREBP2 axis, impairing cholesterol efflux and accelerating VSMCs foam cell formation. Deletion of Dkk1 in VSMCs of *ApoE^−/−^* mice significantly reduced foam cell accumulation and improved plaque stability (106).

Single-cell and spatial transcriptomic analyses have further elucidated the metabolic interactions among ECs, MACs, and VSMCs in human atherosclerotic lesions. One study identified paracrine crosstalk between ITLN1^+^VSMCs-derived foam cells and SPP1^+^MACs, jointly driving inflammation and fibrous cap thinning (107). Additionally, single-cell transcriptomics of human plaques revealed highly integrated metabolic coupling among MACs, and VSMCs, which strongly correlates with plaque stability (108).

In summary, EC metabolic stress initiates a pro-inflammatory environment, MAC metabolic reprogramming sustains foam cell burden and amplifies inflammation, while VSMCs metabolic plasticity governs foam cell expansion and fibrous cap stability. This integrative three-cell metabolic network provides novel therapeutic targets, such as inhibition of EC-PFKFB3, VSMCs-DKK1, or MAC-derived oxysterol pathways. Nevertheless, most evidence to date derives from single-cell type animal models or clinical samples, and direct *in vivo* causal validation of the complete “three-cell circuit” remains lacking, highlighting an important direction for future research.

## 7 Regulation of atherosclerosis by chemically synthesized drugs

### 7.1 ANGPTL3 inhibitors

Angiopoietin-like 3(ANGPTL3) is a protein primarily secreted by the liver and plays a crucial role in lipid metabolism, particularly in the metabolic processes of triglyceride (TG) and low-density lipoprotein cholesterol (LDL-C). Its main mechanism involves inhibiting lipoprotein lipase (LPL) and endothelial lipase (EL). LPL is a key enzyme in lipid metabolism, responsible for converting triglycerides in the blood into fatty acids for the body to use or store (109). EL is mainly involved in the metabolism of HDL and other lipoproteins (110). Research indicates that ANGPTL3 contains an N-terminal coiled-coil domain related to lipid metabolism regulation and a C-terminal fibrinogen-like domain (FLD) related to inflammation. The FLD may play a role in the inflammatory response by binding to integrin αVβ3 (αVβ3) (111). Animal experiments show that during the progression of atherosclerotic (AS) lesions, the expression of αVβ3 in macrophages within the diseased arteries increases. After αVβ3 binds to ANGPTL3, it can cause the continuous activation of NF-κB, increasing the expression of pro-inflammatory cytokines such as tumor necrosis factor-α (TNF-α), interleukin-1β (IL-1β), and interleukin-6 (IL-6) (112). These factors promote the inflammatory response during the formation of foam cells. Therefore, ANGPTL3 inhibitors, by suppressing the activity of ANGPTL3 and blocking the aforementioned mechanisms, attenuate the inflammatory response in macrophages (113) and improve the progression of AS.

### 7.2 PCSK9 inhibitors

As a new type of lipid-lowering drugs, PCSK9 inhibitors have a positive impact on the metabolism associated with AS, and their mechanism of action involves multiple aspects (114).

PCSK9 is primarily expressed in the liver and can bind to the LDLR, promoting the degradation of LDLR. PCSK9 inhibitors can block this binding process, increasing the number of LDLRs on the surface of liver cells. This accelerates the clearance of LDL-C from the blood, significantly reducing its levels, decreasing cholesterol deposition on the vascular wall, and effectively delaying the progression of AS (115). In addition, PCSK9 inhibitors also have an indirect impact on TG and high-density lipoprotein cholesterol (HDL-C). Studies have shown that when LDL-C is reduced, the TG level also decreases, although this effect is relatively weak. For HDL-C, while its content is not directly increased, its reverse cholesterol transport capacity may be enhanced due to the improvement of the overall blood lipid profile, which is beneficial for the progression of AS (116).

Under inflammatory stimulation, inflammatory cells such as monocytes and macrophages are recruited to the vascular intima. PCSK9 inhibitors can not only reduce the generation of oxidized Ox-LDL by lowering blood lipid levels (Ox-LDL can activate endothelial cells and prompt them to express adhesion molecules such as ICAM-1 and VCAM-1, thereby promoting the adhesion and migration of inflammatory cells), but also directly or indirectly inhibit the release of inflammatory factors (such as IL-1, IL-6, TNF-α, etc.) (117, 118). Moreover, it has been found that PCSK9 inhibitors may interfere with inflammatory signaling pathways such as NF-κB, inhibiting the activation of NF-κB and reducing the expression of inflammatory factor genes, thereby alleviating vascular inflammatory reactions and reducing the promoting effect of inflammation on AS (119).

## 8 Regulation of atherosclerosis by Chinese patent medicines

### 8.1 Tongxinluo capsule

Tongxinluo (TXL) is a Chinese patent medicine approved in 1996 for treating atherosclerosis and angina pectoris. It contains 12 animal and plant ingredients, including ginseng, leeches, and scorpions. Research by G. Q. Yuan's team showed that TXL can significantly reduce total cholesterol and LDL-C levels, stabilize atherosclerotic plaques, and decrease serum cholesterol. The mechanism involves downregulating LLP and Ox-LDL receptor expression, reducing cholesterol uptake and depositionl (120, 121). TXL also upregulates ABCA1, enhancing reverse cholesterol transport and lowering intracellular cholesterol (122). Xuejiao Jiang's team found that TXL can alleviate Ox-LDL-induced damage to endothelial cells, reverse the high expression of proteins like GSDMD, and synergize with the caspase-1 inhibitor VX-765. It reduces ROS accumulation and inhibits the NLRP3/caspase-1 signaling pathway, decreasing inflammatory factor release (121).

In atherosclerotic rat models, TXL treatment significantly downregulated serum levels of ET-1, MCP-1, and sICAM-1, while increasing NO levels. It also downregulated ICAM-1 and MCP-1 expression in damaged arteries, indicating improved endothelial function and reduced inflammation, thus delaying atherosclerosis progression (123). TXL can regulate vascular endothelial cell junction proteins, enhance endothelial barrier function, activate the ERK1/2 signaling pathway, and improve endothelial function by tightening tight junctions and reducing vascular permeability (124). These experiments show that TXL has multi-target therapeutic effects on atherosclerosis, significantly improving its pathological process.

### 8.2 Xuezhikang

Xuezhikang (XZK) is a natural medicine derived from red yeast rice, containing statin homologues like lovastatin, unsaturated fatty acids, plant sterols, and flavonoids. It mildly inhibits cholesterol synthesis, reducing small LDL-C and improving insulin resistance. XZK significantly lowers LDL-C, TC, and TG levels while raising HDL-C (125). It also decreases plasma Ox-LDL, reducing oxidative stress and inhibiting AS development (126, 127).

At the vascular endothelial cell level, XZK up-regulates eNOS expression. In animal studies, XZK-treated AS rats show higher eNOS expression in aortic endothelial cells than controls. XZK also promotes eNOS expression in red blood cells and increases plasma NOx levels. By down-regulating caveolin-1 in the aortic wall, XZK reduces eNOS inhibition, enhancing vascular endothelial function. These actions collectively support vascular health and aid in preventing and treating cardiovascular diseases (128).

## 9 Emerging therapeutic approaches

Nanomedicine is increasingly showing promise as a complement to traditional therapies for atherosclerosis by enabling targeted delivery, controlled release, and modulation of plaque microenvironments. For example, black phosphorus quantum dots (BPQDs) have been administered every other day for 3 weeks via intravenous injection in high-fat diet *ApoE^−/−^* mice. BPQDs promoted autophagy in macrophage foam cells, improved lipid metabolism, decreased serum lipid levels, increased vascular elasticity, and thus prevented AS development more effectively than simvastatin in that model (129).

Another strategy uses ROS-responsive nanoprodrugs: a redox-responsive simvastatin prodrug (TPTS) linked via a thioketal linker was used to form nanoparticles that release simvastatin in response to oxidative stress. *in vivo*, this system reduced inflammatory cytokines (TNF-α, IL-1β, MCP-1), inhibited macrophage M1 polarization, decreased ROS levels, and yielded superior anti-atherosclerotic effects compared with free drug controls (130).

Also, biomimetic, ROS-responsive hyaluronic acid–based nanoparticles loaded with methotrexate and coated with macrophage membrane (MM/MTXNPs) have been shown to accumulate in plaques, evade macrophage phagocytosis, reduce foam cell formation *in vitro*, and inhibit plaque development in *ApoE^−/−^* mice more effectively than non-coated versions or free drug (131).

Moreover, a bionic nano-delivery platform combining platelet membrane–coated black phosphorus nanosheets (BPNSs) with siRNA against CaMKIIγ was used in *ApoE^−/−^* mice: this system scavenged ROS in macrophages, restored efferocytosis via upregulation of MerTK, cleared apoptotic cells, improved plaque stability, and largely inhibited AS progression (132).

Compared with established treatments such as PCSK9 and ANGPTL3 inhibitors or Chinese patent medicines like Tongxinluo and Xuezhikang, emerging nanomedicine strategies offer distinct advantages in targeting and metabolic regulation. Traditional therapies primarily act through systemic lipid-lowering and anti-inflammatory effects to improve vascular function, whereas nanocarriers enable precise drug delivery, controlled release, and local modulation of macrophage and smooth muscle cell metabolism within plaques, thereby alleviating inflammation and oxidative stress simultaneously. These systems exhibit prolonged efficacy, reduced systemic toxicity, and promote metabolic reprogramming at the cellular level. In the future, combining nanodelivery platforms with conventional agents may represent a promising direction for personalized, metabolism-targeted therapy in atherosclerosis.

## 10 Conclusion and perspective

In summary, this article explores the role of dysregulated cellular metabolism in AS, emphasizing that metabolic alterations in macrophages, ECs, and VSMCs profoundly influence disease progression and offer new therapeutic insights. Among these, lipid metabolic disturbances-including cholesterol accumulation, fatty acid oxidation imbalance, and triglyceride-rich lipoprotein-induced endothelial dysfunction-constitute a central driver linking inflammation and phenotypic transformation during plaque development.

Future research should further investigate how metabolic reprogramming regulates various forms of programmed cell death, such as ferroptosis, cuproptosis, and disulfidptosis, as well as the contributions of vitamin metabolism and gut microbiota–derived metabolites to AS pathogenesis. Traditional Chinese medicines such as Tongxinluo capsules, Xuezhikang, and Shexiang Baoxin pills have demonstrated potential in modulating lipid and energy metabolism, yet the metabolic effects of other formulations remain to be elucidated.

Collectively, these directions highlight the importance of targeting metabolic pathways as a core strategy for understanding, preventing, and treating atherosclerosis.
